# Evidence That *GRIN2A* Mutations in Melanoma Correlate with Decreased Survival

**DOI:** 10.3389/fonc.2013.00333

**Published:** 2014-01-13

**Authors:** Stacey Ann N. D’mello, Jack U. Flanagan, Taryn N. Green, Euphemia Y. Leung, Marjan E. Askarian-Amiri, Wayne R. Joseph, Michael R. McCrystal, Richard J. Isaacs, James H. F. Shaw, Christopher E. Furneaux, Matthew J. During, Graeme J. Finlay, Bruce C. Baguley, Maggie L. Kalev-Zylinska

**Affiliations:** ^1^Department of Molecular Medicine and Pathology, School of Medical Sciences, University of Auckland, Auckland, New Zealand; ^2^Auckland Cancer Society Research Centre, University of Auckland, Auckland, New Zealand; ^3^Maurice Wilkins Centre for Molecular Biodiscovery, University of Auckland, Auckland, New Zealand; ^4^Department of Clinical Oncology, Auckland District Health Board, Auckland, New Zealand; ^5^Canopy Cancer Care, Mercy Hospital, Auckland, New Zealand; ^6^Regional Cancer Treatment Service, Palmerston North Public Hospital, Palmerston North, New Zealand; ^7^Oncology Surgery Ltd., Auckland, New Zealand; ^8^Department of Neurosurgery, Auckland District Health Board, Auckland, New Zealand; ^9^Department of Molecular Virology, Immunology and Medical Genetics, Neuroscience and Neurological Surgery, Ohio State University, Columbus, OH, USA; ^10^Centre for Brain Research, University of Auckland, Auckland, New Zealand; ^11^LabPlus Haematology, Auckland District Health Board, Auckland, New Zealand

**Keywords:** melanoma, *GRIN2A*, mutation, GluN2A, NMDAR, NMDA receptor, glutamate, prognosis

## Abstract

Previous whole-exome sequencing has demonstrated that melanoma tumors harbor mutations in the *GRIN2A* gene. *GRIN2A* encodes the regulatory GluN2A subunit of the glutamate-gated *N*-methyl-d-aspartate receptor (NMDAR), involvement of which in melanoma remains undefined. Here, we sequenced coding exons of *GRIN2A* in 19 low-passage melanoma cell lines derived from patients with metastatic melanoma. Potential mutation impact was evaluated *in silico*, including within the GluN2A crystal structure, and clinical correlations were sought. We found that of 19 metastatic melanoma tumors, four (21%) carried five missense mutations in the evolutionarily conserved domains of *GRIN2A*; two were previously reported. Melanoma cells that carried these mutations were treatment-naïve. Sorting intolerant from tolerant analysis predicted that S349F, G762E, and P1132L would disrupt protein function. When modeled into the crystal structure of GluN2A, G762E was seen to potentially alter GluN1–GluN2A interactions and ligand binding, implying disruption to NMDAR functionality. Patients whose tumors carried non-synonymous *GRIN2A* mutations had faster disease progression and shorter overall survival (*P* < 0.05). This was in contrast to the *BRAF* V600E mutation, found in 58% of tumors but showing no correlation with clinical outcome (*P* = 0.963). Although numbers of patients in this study are small, and firm conclusions about the association between *GRIN2A* mutations and poor clinical outcome cannot be drawn, our results highlight the high prevalence of *GRIN2A* mutations in metastatic melanoma and suggest for the first time that mutated NMDARs impact melanoma progression.

## Introduction

The genomic revolution of recent years has led to substantial advances in the cataloging of mutations in melanoma, most notable of which have been activating mutations in *BRAF*, *NRAS*, and *KIT* genes (1). The presence of these mutations helps to guide treatment with RAF, MEK, and KIT inhibitors, but they do not predict disease progression or survival of patients (2). In general, the usefulness of molecular biomarkers in determining prognosis in melanoma remains limited.

Mutations in *GRIN2A* have been reported in up to a third of melanoma samples (3), although there is wide variation between studies (4–7) and no data on their clinical relevance. The *GRIN2A* gene, located on chromosome 16p13.2, encodes the GluN2A protein, a regulatory subunit of the glutamate-gated *N*-methyl-d-aspartate receptor (NMDAR) (8, 9). NMDARs are best known for their roles in the brain; hence, the finding of *GRIN2A* mutations in melanoma had been unexpected. Nevertheless, NMDARs have attracted attention for their potential contribution in cancer due to the effects on cell death, survival, and migration (10, 11). Excessive NMDAR activation overloads the cell with calcium and leads to cell death (12). On the other hand, normal NMDAR activity promotes cell survival through the phosphatidylinositol 3-kinase (PI3-K) and extracellular signal-regulated kinase (ERK) signaling pathways (13). In addition, NMDAR effects on cell migration may affect tumor spread in tissue (10). Current knowledge on the NMDARs in the context of melanoma is limited, although expression of GluN2A in both normal and malignant melanocytes has been demonstrated (14, 15). NMDAR inhibitors reduce migration and proliferation of melanoma cells *in vitro* (15).

In response to the previously published exome sequencing data (3), we have investigated the prevalence of *GRIN2A* mutations in 19 low-passage metastatic melanoma cell lines out of over a 100 developed in our institution, and retrospectively correlated the presence of *GRIN2A* mutations with patient outcome.

## Materials and Methods

### Patients and tumor material

Low-passage melanoma cell lines derived from 19 patients with metastatic melanoma treated at two independent national sites were used in this study (Table S1 in Supplementary Material). Written informed consent was obtained from all participants prior to enrolment; all study procedures were approved by Northern A Health and Disability Human Ethics Committee. This was not a clinical trial, and study procedures did not affect patient management in any way. Patients underwent skin, lymph node, or distant organ biopsies for diagnosis, staging, or treatment, as required clinically. Excess tissue was used to establish melanoma cell lines, as described (16). Cell lines for sequencing were chosen randomly out of over a 100 previously established in our center; all were passaged <30 times. Cells were grown in 25 or 75 cm^2^ flasks to 75% confluency in a low-oxygen humidified incubator (37°C, 5% O_2_, and 5% CO_2_ in nitrogen). Cultures were maintained in Alpha-modified Minimal Essential Medium (Sigma-Aldrich, Saint Louis, MO, USA) containing 5% fetal bovine serum (Life Technologies, Carlsbad, CA, USA), penicillin (100 units ml^−1^), streptomycin (100 μg ml^−1^) (Sigma-Aldrich), insulin (5 μg ml^−1^), transferrin (5 μg ml^−1^), and sodium selenite (5 ng ml^−1^) (Roche Applied Science, San Diego, CA, USA). Melanoma cells were sub-cultured weekly to maintain them in a proliferative state.

Normal human epidermal melanocytes were used as control cells (HEMa-LP, Life Technologies); we did not have access to non-tumor tissue from patients. Melanocytes were cultured in Medium 254 (M-254-500; Life Technologies) supplemented with Human Melanocyte Growth Supplement (Life Technologies). Cultures were maintained in a humidified incubator (5% CO_2_ in air) at 37°C. Melanocytes were sub-cultured every 4 weeks.

### Sequencing

DNA was isolated using a PureLink Genomic DNA kit (Life Technologies), according to the manufacturer’s instructions. Concentrations and purity of DNA were determined using a nanodrop spectrophotometer (ND-1000, NanoDrop, ThermoFisher Scientific, Rockford, IL, USA). Twelve coding exons of *GRIN2A* (numbered 3–14), including their flanking intronic regions, were sequenced using Sanger method. The sequencing primers were as previously reported (3), except for 7Reverse (5′-GCAGGCCCTTTGTCTGAGTA-3′) and 8Forward (5′-CCTTGCATCCAGGTGGTC-3′), which we designed using Primer3web version 4.0.0 software^1^ to reduce interference from polyA sequences located in the intron between *GRIN2A* exons 7 and 8. PCR conditions for all primers are provided in Table 1. PCR reactions were performed in a final volume of 25 μl 1× PCR buffer containing 50–100 ng DNA, 0.3 μM forward and reverse primers each, 5 U Expand High Fidelity Enzyme mix (Roche Applied Science), 0.2 mM deoxynucleoside triphosphates, and 1.5 mM MgCl_2_. Bovine serum albumin (3 ng μl^−1^; Life Technologies) was used to counteract PCR inhibition where required. The correct sizes of the amplicons were confirmed on 2% agarose gels with DNA visualized with GelRed (Biotium, Hayward, CA, USA). DNA concentrations were estimated against Low-Mass Ladder (Life technologies). Sequencing was performed in two directions using ABI Prism 3730xl Genetic Analyzer with the ABI PRISM Big Dye Terminator Cycle Sequencing Ready Reaction kit, version 3.1 (Applied Biosystems, Foster City, CA, USA).

**Table 1:** **PCR cycling conditions used to amplify *GRIN2A* sequences**.

Amplicon name	Amplicon size (bp)	Denaturation	Annealing	Extension	Cycles number
*GRIN2A*_1 and 2	710	94°C, 30 s	66°C, 30 s	72°C, 45 s	
*GRIN2A*_3 and 4	763	94°C, 30 s	60°C, 30 s	72°C, 30 s	
*GRIN2A*_5	331	94°C, 30 s	60°C, 30 s	72°C, 30 s	
*GRIN2A*_6	464	94°C, 30 s	60°C, 30 s	72°C, 30 s	
*GRIN2A*_7	350	94°C, 30 s	60°C, 30 s	72°C, 30 s	
*GRIN2A*_8	295	94°C, 30 s	60°C, 30 s	72°C, 30 s	
*GRIN2A*_9	362	94°C, 30 s	68°C, 30s	72°C, 30 s	35
*GRIN2A*_10	428	94°C, 30 s	60°C, 30 s	72°C, 30 s	for all
*GRIN2A*_11	372	94°C, 30 s	60°C, 30 s	72°C, 30 s	
*GRIN2A*_12	373	94°C, 30 s	60°C, 30 s	72°C, 30 s	
*GRIN2A*_13	381	94°C, 30 s	60°C, 30 s	72°C, 30 s	
*GRIN2A*_14	397	94°C, 30 s	60°C, 30 s	72°C, 30 s	
*GRIN2A*_15 and 16	633	94°C, 30 s	60°C, 30 s	72°C, 45 s	
*GRIN2A*_17 and 18	759	94°C, 30 s	60°C, 30 s	72°C, 45 s	
*GRIN2A*_19 and 20	758	94°C, 30 s	60°C, 30 s	72°C, 45 s	

The *BRAF* V600E mutation status had been determined previously for most cell lines (17). For the remaining samples, Cobas^®^ 4800 *BRAF* Mutation Test (Roche Molecular Systems, Pleasanton, CA, USA) was used.

### Sequence analysis and prediction of mutation impact

Exon sequences of *GRIN2A* were analyzed by reference to human *GRIN2A* (GenBank accession number NG_011812; RefSeqGene number GI: 226492187) using Geneious Pro 5.6.4 software (Biomatters, Auckland, New Zealand). To help predict if amino acid substitutions would affect protein function, Sorting Intolerant from Tolerant (SIFT) analysis of mutations was performed^2^ (18), applying UniProt SWISS-PROT 57.15 database. Catalog of somatic mutations in cancer (COSMIC)^3^ and MelanomaDB (19) databases were interrogated to search for *GRIN2A* mutations previously found in melanoma. Common germline single nucleotide polymorphisms (SNPs) in *GRIN2A* were eliminated using the NCBI database of SNPs^4^ (20).

### Modeling mutation impact

The G762E mutation was modeled into the X-ray crystal structure of the GluN1–GluN2A S1S2 heterodimer using Modeller 9.1 (21) and the Protein Data Bank entry 2A5T (22) as the template. Ten models were constructed using two rounds of optimization with the slow autoschedule and molecular dynamics refinement options; all other settings were kept at default values. The models were superimposed on the 2A5T heterodimer using PyMol (23) and inspected visually.

### Collection of clinical data and statistical analysis

Data on antecedent primary melanoma, disease progression, and treatment were obtained from a retrospective review of patient medical records. Melanoma staging was determined clinically based on the criteria designated by the American Joint Committee on Cancer (24). In this system, patients with lymph node metastases are designated as stage III; stage IV disease is defined by the presence of distant organ metastases. Overall survival is shown as the length of time from the initial diagnosis of melanoma to death. Considering that patient groups were small in this study, we presented quantitative data as median (range) (Table 2) to best demonstrate sample distribution. To compare groups, cross-tabulations with significance tests were performed for data in categories. Analysis for mean differences between groups was performed using one-way ANOVA. Differences in time to event data were analyzed for the effect of *GRIN2A* mutation status using the survival analysis method (log-rank test). Kaplan–Meier curves were generated to plot time to events (such as development of lymph node or distant organ metastases, or death) for individual patients (dot plots are provided as Supplemental Material). Statistical analysis was conducted using IBM SPSS Statistics software package for Windows, version 19.0 (Chicago, IL, USA) (25). *P* values <0.05 were considered statistically significant.

**Table 2:** **Clinical characteristics for all patients, according to the presence or absence of *GRIN2A* mutations in tumor-derived cell lines**.

	All patients	Non-synonymous *GRIN2A* mutations	Synonymous *GRIN2A* mutations only	Non-mutated *GRIN2A*	*P* value
	*n* = 19	*n* = 4	*n* = 3	*n* = 12	
Age: median (range) years	54 (36–81)	58 (36–69)	46 (40–69)	56 (38–81)	0.916^a^
Males *n* (%)	13 (68)	4 (100)	1 (33)	8 (67)	0.168^b^
Duration of known melanoma prior to enrolment: median (range) months	31 (0–211)	14 (0–30)	6 (4–56)	88 (1–211)	0.110^a^
Patients with distant metastases at enrolment: *n* (%)^c^	12 (63)	1 (25)	2 (67)	9 (75)	0.198^b^
Patients treated with chemotherapy: *n* (%)	5 (26)	0	2 (67)	3 (25)	0.149^b^
Patients treated with autologous tumor vaccine: *n* (%)	3 (16)	0	1 (33)^d^	2 (17)	0.492^b^
Progression from diagnosis to lymph node metastases (stage III): median (range) months	9 (0–140)	0 (0–27)	6 (0–17)	37 (0–140)	0.040^e^
Progression from diagnosis to distant organ metastases (stage IV): median (range) months	34 (0–205)	2 (0–34)	14 (4–35)	108 (1–205)	0.012^e^
Progression from diagnosis to death (i.e. overall survival): median (range) months	36 (4–229)	5 (4–36)	15 (5–61)	114 (4–229)	0.013^e^

*“All patients” column demonstrates patient characteristics at enrolment into the study. The *P* values are for the effect of *GRIN2A* mutation status, so the data in the last three columns were used for statistical analysis (i.e. excluding the “All patients” column)*.

^a^ One-way ANOVA

^b^ χ^2^ test

*^c^ Other patients had lymph node metastases at enrolment*.

*^d^ This patient also received chemotherapy*.

*^e^ Log-rank *P* values reflect differences between patients with non-mutated *GRIN2A* versus patients with non-synonymous *GRIN2A* mutations. When patients with synonymous *GRIN2A* mutations were compared, differences were not statistically significant (pairwise *P* values are shown in Figures 3 and 4 and Figure S1 in Supplementary Material)*.

## Results

Nineteen low-passage melanoma cell lines, derived from 19 patients with metastatic melanoma, were used in this study to sequence coding exons of *GRIN2A*, together with their flanking intronic regions. Patient characteristics at enrolment into the study are shown in Table 2 (“All patients” column). Apart from two patients who presented with either bulky or disseminated disease and entered this study on presentation, other patients had a prior history of skin melanoma dating back a median of 34 (1–211) months. Seventeen patients had primary skin lesions in sun-exposed areas; in the other two, primary skin lesions remained occult. At the time of enrolment (between 1989 and 2010), 12 patients (63%) had metastatic melanoma in distant organs (stage IV disease) and 7 patients (37%) had lymph node involvement (stage III disease). All patients were managed surgically and with regional radiotherapy. Five patients also received chemotherapy – three had POC (procarbazine, vincristine, and lomustine), one temozolomide, and one DTIC (dacarbazine) – with the number of chemotherapy cycles ranging from one to six. Autologous tumor vaccine was used in three patients (in one, after an unsuccessful course of chemotherapy). One patient also received an experimental vascular disrupting agent within a phase I clinical trial, as well as interferon α. No patient received BRAF inhibitors. Treatment was the decision of a clinical team, independent of research procedures. One patient remained free of melanoma 12 years after nodal recurrence. The other 18 patients have all died of their disease. The median survival time was 36 (4–229) months.

### *GRIN2A* mutations and predicted impact on protein structure

Sequencing of *GRIN2A* was conducted using cell lines derived from tumors spread to lymph nodes in 12 patients (63%), distant organs (brain, small bowel, ascites, or lung) (five patients; 26%), or locoregional metastases (two patients; 11%). All cell lines used in this study were passaged <30 times, and were generated from treatment-naïve tumors, except for NZM 017 and NZM 040, which were derived from patients who received prior immunotherapy or chemotherapy, respectively (Table S1 in Supplementary Material).

**Figure 1:**
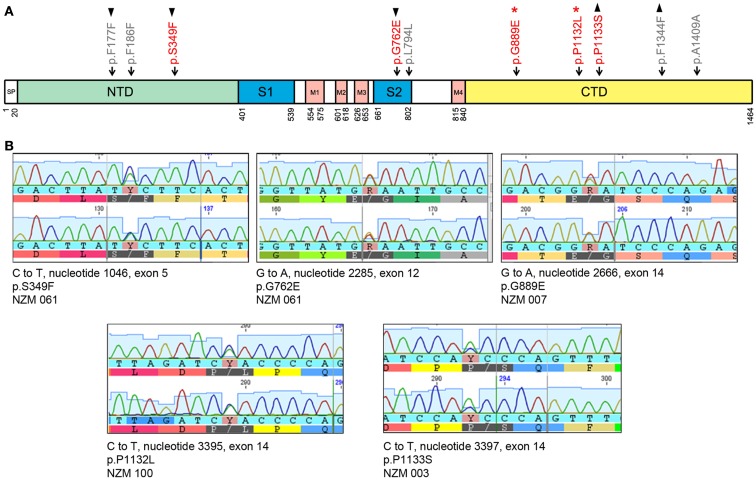
***GRIN2A* mutations in melanoma cell lines**. **(A)** Schematic of the GluN2A protein together with the non-synonymous (in red) and synonymous (in gray) mutations in *GRIN2A*. *GRIN2A* cDNA is 4392 nucleotides long and can be divided into sections encoding evolutionarily conserved domains in the GluN2A protein. The first and last amino acid residues of the SP, S1, S2, and M1–M4 domains are numbered. Symbols ▴ and ▾ mark mutations that coincided in the same tumor samples; *marks mutations reported previously. Abbreviations: SP, signal peptide; NTD, N-terminal domain; S1 and S2 segments form the glutamate-binding domain; M1–M4 transmembrane segments form the ion channel pore; CTD, C-terminal domain. **(B)** Sanger sequencing output of non-synonymous mutations in *GRIN2A*. Each panel represents a non-synonymous mutation detected. Nucleotides are labeled below their respective peaks. Corresponding amino acid sequences are indicated.

Of 19 tumor samples tested, four (21%) carried five non-synonymous mutations in *GRIN2A* (Figure 1; Table 3). Tumors that carried these mutations were treatment-naïve, excluding therapy effect. All non-synonymous mutations were missense: three clustered in exon 14 (G889E, P1132L, P1133S), and the other two in exon 5 (S349F) and exon 12 (G762E) (Figure 1). These locations corresponded with the evolutionarily conserved domains in the GluN2A protein: C-terminal, N-terminal, and the S2 segment, respectively (Figure 1). The S2 segment forms the ligand binding domain, and the intracytoplasmic C-terminus is involved in intracellular signaling and interactions with the cytoskeleton. Two mutations, G889E and P1132L, were previously reported [Ref. (3, 17), respectively]. SIFT analysis predicted that S349F, G762E, and P1133S would deleteriously affect protein function (Table 3).

**Table 3:** **Non-synonymous mutations in *GRIN2A***.

NZM cell line	Substitution and nucleotide number	Amino acid change	Exon	Zygosity	SIFT score	SIFT median
061	C > T	S349F	5	Hetero-	0	3.33
	c.1046	
	g.291693	
061	G > A	G762E	12	Hetero-	0	3.08
	c.2285	
	g.384407	
007	G > A	G889E	14	Hetero-	0.29	3.24
	c.2666	
	g.417877	
100	C > T	P1132L	14	Hetero-	0.2	3.32
	c.3395	
	g.418606	
003	C > T	P1133S	14	Hetero-	0	3.32
	c.3397	
	g.418608	

*Mutations are listed in the order of location along the sequence. Deleterious substitutions were predicted from SIFT scores ≤0.05*.

*c, cDNA; g, genomic DNA; hetero-, heterozygous; SIFT, sorting intolerant from tolerant analysis*.

Five synonymous mutations were also detected (Table 4), as well as four SNPs (Table 5); SNPs were excluded from further analysis. F186F synonymous mutation was found in the NZM 040 cell line derived from patient who received two cycles of POC chemotherapy 8 months prior to cell line derivation (Table S1 in Supplementary Material). In this instance, the possibility that therapy contributed to the presence of this mutation could not be ruled out.

**Table 4:** **Synonymous mutations in *GRIN2A***.

NZM cell line	Substitution and nucleotide number	Amino acid	Exon	Zygosity
061	C > T	F177F	4	Hetero-
	c.531	
	g.244320	
040	C > T	F186F	4	Hetero-
	c.558	
	g.244347	
055	C > T	L794L	13	Homo-
	c.2380	
	g.413689	
003	C > T	F1344F	14	Hetero-
	c.4032	
	g.419243	
086	A > C	A1409A	14	Hetero-
	c.4227	
	g.419438	

*Mutations are listed in the order of location along the sequence*.

*c, cDNA; g, genomic DNA; hetero-, heterozygous; homo-, homozygous*.

**Table 5:** ***GRIN2A* SNPs in tumor-derived cell lines**.

NZM cell line	Substitution and nucleotide number	Amino acid	Exon	Zygosity
011	G > A	L425L	6	Homo-
007	c.1275	
055	g.332946	
001	G > A	L425L	6	Hetero-
034	c.1275	
061	g.332946	
011	G > C	R695R	11	Homo-
007	c.2085	
055	g.360408	
01	G > C	R695R	11	Hetero-
061	c.2085	
	g.360408	
006	C > T	W730W	12	Homo-
	c.2190	
	g.384312	
003	C > A	N1076K	14	Hetero-
	c.3228	
	g.418439	

*c, cDNA; g, genomic DNA; homo-, homozygous; hetero-, heterozygous*.

**Figure 2:**
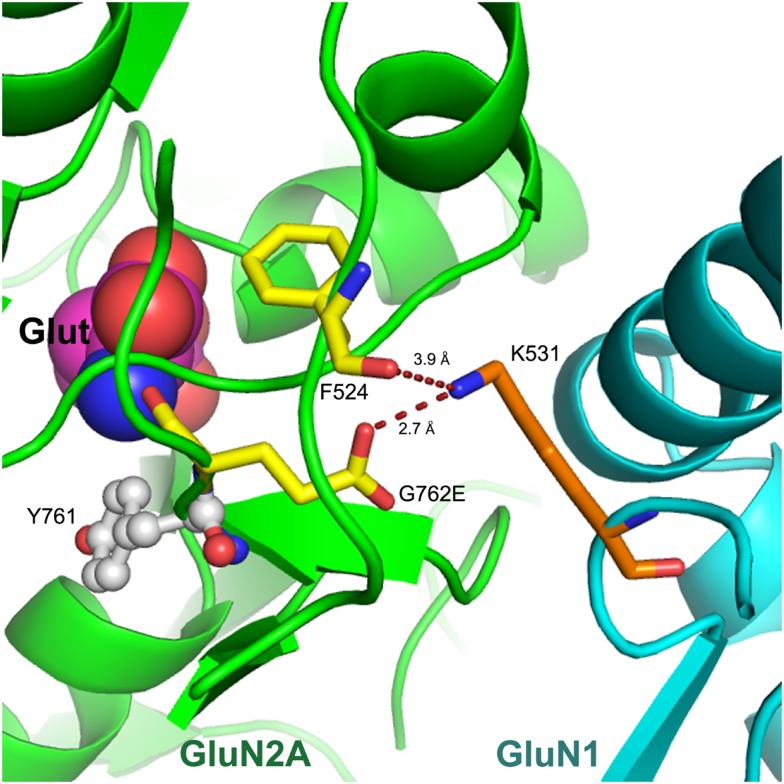
**Model of G762E within the GluN1–GluN2A X-ray crystal structure**. The S1S2 loop of GluN2A (in green) is shown interfacing with GluN1 (in cyan). A portion of the GluN2A agonist-binding site is on the left together with bound glutamate (Glut). G762E and F524 residues of GluN2A are 2.7 and 3.9 Å away from K531 in GluN1, respectively; potential new electrostatic interactions are indicated as dashed lines. Nitrogen atoms are in blue, and oxygen in red.

We modeled the G762E missense substitution into the 3-dimensional X-ray crystal structure of the GluN1–GluN2A heterodimer, 2A5T (22) (Figure 2). This revealed that G762E was located in the distal “hinge” of the glutamate-binding clam-shell-like region of GluN2A. In this location, the mutated glutamate residue was seen to interact with K531 in the GluN1 protein interfacing GluN2A in this region. While K531 formed a hydrogen bond with the backbone carbonyl of F524 in GluN2A, its proximity to the mutated glutamate side-chain (G762E) indicated the potential for new electrostatic interactions between G762E (in GluN2A) and K531 (in GluN1) (Figure 2) with the ability to alter interactions between GluN2A and GluN1 subunits and consequently, impact NMDAR functionality. Conformational changes that developed to accommodate G762E could also affect ligand binding, as the residue preceding G762E (Y761) was part of the glutamate-binding site (Figure 2) (26).

### Clinical associations of *GRIN2A* mutations

**Figure 3:**
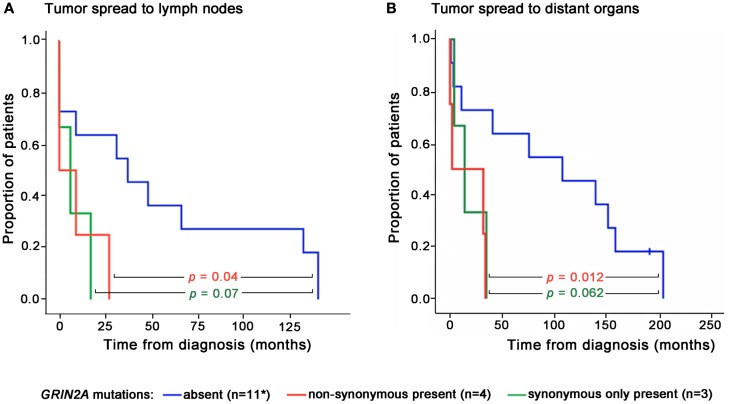
**Times of disease progression from diagnosis to lymph node (A) or distant organ (B) metastases for individual patients according to the presence or absence of *GRIN2A* mutations**. Levels of statistical significance are shown. *Progression data for one patient with non-mutated *GRIN2A* was not available.

**Figure 4:**
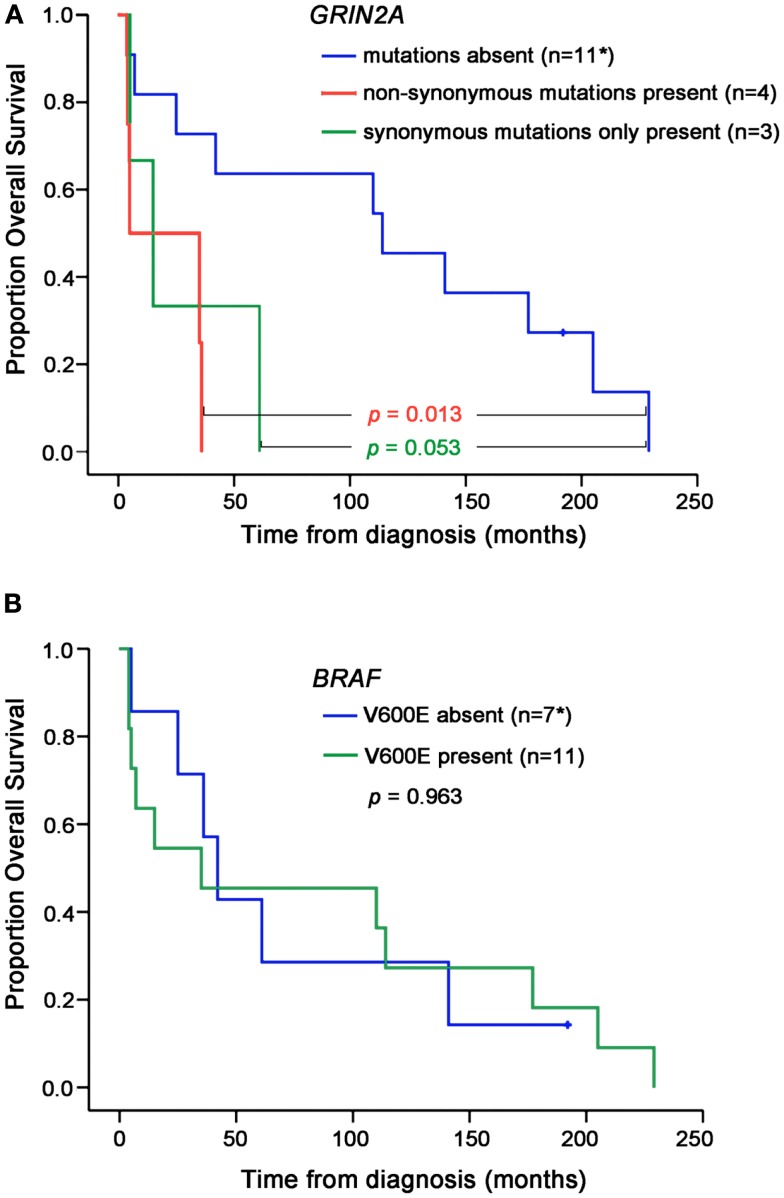
**Overall survival according to the *GRIN2A* (A) or *BRAF* V600E (B) mutation status**. Levels of statistical significance are shown. *Data for one patient with non-mutated *GRIN2A* (V600E absent) was not available.

There was no difference in age and gender between patients whose melanoma lines carried *GRIN2A* mutations and those who did not (Table 2). Two patients in this study presented with disseminated melanoma and both were found to carry non-synonymous mutations in *GRIN2A*. The other two patients with the non-synonymous *GRIN2A* mutations presented with skin lesions that spread to lymph nodes within 9 and 27 months, compared with a median of 37 (0–140) months for patients with non-mutated *GRIN2A* (*P* = 0.041; Table 2). Overall, patients with non-synonymous *GRIN2A* mutations had faster progression of melanoma from skin lesions to the involvement of lymph nodes (*P* = 0.04) or distant organs (*P* = 0.012), and shorter overall survival (*P* = 0.013) compared with patients with non-mutated *GRIN2A* (Table 2; Figures 3 and 4A). The *BRAF* V600E mutation was found in 11 of 19 (58%) tumor samples but in contrast to *GRIN2A*, its presence showed no correlation with overall survival (*P* = 0.963; Figure 4B; Table S1 in Supplementary Material).

Seven patients in this study received systemic therapy – five had chemotherapy and another two autologous tumor vaccine (Table 2). These were in addition to surgery and involved field radiotherapy used in all patients. When compared with patients managed with surgery and radiotherapy alone, the administration of systemic therapy did not change disease spread to lymph nodes (*P* = 0.849), distant organs (*P* = 0.499), or overall survival (*P* = 0.843).

Patients with isolated synonymous *GRIN2A* mutations (*n* = 3) displayed a trend for poorer outcome, compared with patients with non-mutated *GRIN2A*, but this was not statistically significant (Figures 3 and 4).

## Discussion

This study has shown that cell lines derived from 4 of 19 (21%) patients with metastatic melanoma carried five missense mutations in *GRIN2A*. They occurred in three of the four evolutionarily conserved domains of the GluN2A subunit of the NMDAR: the N-terminal, glutamate-binding, and C-terminal domains. All missense mutations were found in treatment-naïve samples. The S349F, G762E, and P1132L substitutions were predicted by SIFT to disrupt protein function. When modeled into the crystal structure of GluN2A, the G762E substitution was seen to potentially alter GluN1–GluN2A interactions and ligand binding, implying disruption to the NMDAR functionality. Patients whose tumors carried non-synonymous mutations in *GRIN2A* had faster disease progression and shorter overall survival. Our findings suggest for the first time, to our knowledge, that *GRIN2A* mutations may drive melanoma progression.

Our results are in agreement with the seminal whole-exome sequencing work, where non-synonymous mutations in *GRIN2A* were found in 26% of melanoma samples (3). Other exome-wide sequencing projects detected *GRIN2A* mutations at lower frequencies (4–7). The true frequency of *GRIN2A* mutations in melanoma remains uncertain, but our results support the high prevalence of *GRIN2A* mutations in metastatic melanoma (3). Possible causes for differences between studies include the stage of tumors tested, or biological heterogeneity of tumors influenced by demographic, geographical, or environmental factors. Our study demonstrates for the first time that patients with *GRIN2A* mutations may have more aggressive disease. Considering that there is currently no reliable genetic biomarker that predicts melanoma progression, *GRIN2A* mutation testing may offer valuable prognostic information. Earlier detection of highly aggressive tumors could assist faster introduction of new therapies for melanoma patients. We propose that the *GRIN2A* mutation testing be incorporated in larger prospective studies for further evaluation of our findings.

Our work has obvious limitations. Patient numbers are small, and the clinical outcome was assessed retrospectively. We did not have access to non-diseased patient DNA to exclude germline polymorphisms (SNPs were excluded using online databases). Original tumor tissue is no longer available to confirm that these contained *GRIN2A* mutations found in cell lines, but an error in cell line authentication is extremely unlikely. Short tandem repeat profiling has been conducted on NZM cell lines to ensure authentication is possible in the future; however, profiles of original tumors are not available. Small sample size limits our conclusions, and confounders cannot be excluded. Nevertheless, the *BRAF* mutation status and systemic therapy had no effect on clinical outcome in these patients. Other possible limitations include the relatively late stage of patients at presentation and the selection of cell lines tested. Our success rate of establishing melanoma cell lines is at least 90%, but the possibility of bias toward melanomas that can be grown in culture cannot be ruled out.

Synonymous mutations in *GRIN2A* associated with poorer patient outcome; however, these observations were not statistically significant. Recent studies indicate that synonymous mutations may be important in cancer, primarily through mechanisms that affect RNA processing and protein translation (27, 28). Further work in this area will be required to clarify if synonymous *GRIN2A* mutations play a role in melanoma biology.

Our results have strong implications for basic research. The roles of NMDAR-mediated pathways in melanoma are still unknown and will require elucidation. Well-characterized melanoma cell lines with known mutations, such as those described in this manuscript, will be valuable tools to examine the mechanisms of action and consequences of specific *GRIN2A* mutations in melanoma tumors. We hypothesize that possible mechanisms through which G762E and other *GRIN2A* mutations interfere with the NMDAR include reduced NMDAR channel function and disturbed intracellular signaling downstream. Such effects would be most relevant under conditions of NMDAR overactivation, where excessive calcium uptake induces cell toxicity. The lack of NMDAR-mediated cell death could facilitate tumor progression. Our hypothesis is consistent with the previously suggested role for the NMDAR as a tumor suppressor (29). Other GluN subunits (if expressed in melanoma cells) could compensate for the GluN2A disruption or contribute additional functionality. NMDAR-mediated pro-cell-survival signaling could also provide oncogenic effects, in keeping with the functional dichotomy of the NMDAR (11).

In conclusion, our study suggests that non-synonymous mutations in *GRIN2A* are present in approximately 20% of patients with metastatic melanoma and associate with faster disease progression and shorter overall survival. The most direct clinical implication of our work is that *GRIN2A* mutation status may allow earlier detection and hence faster treatments of patients with aggressive tumors. Our data also imply that NMDAR may be a novel molecular modifier in melanoma; hence, further studies into its biological role should be pursued.

## Author Contributions

Stacey Ann N. D’mello conducted experimental work, analyzed data, and wrote the manuscript together with Maggie L. Kalev-Zylinska; Jack U. Flanagan supervised structural modeling of mutation impact; Taryn N. Green, Euphemia Y. Leung, Marjan E. Askarian-Amiri, and Wayne R. Joseph provided advice and assisted experimental procedures; Michael R. McCrystal provided clinical advice; Richard J. Isaacs, James H. F. Shaw, and Christopher E. Furneaux contributed patient samples; Matthew J. During provided mentorship and advice; Graeme J. Finlay provided supervision and edited the manuscript; Bruce C. Baguley helped to design the study, provided supervision, and advice; Maggie L. Kalev-Zylinska designed the study, obtained and analyzed clinical data, provided supervision, and wrote the manuscript together with Stacey Ann N. D’mello.

## Conflict of Interest Statement

The authors declare that the research was conducted in the absence of any commercial or financial relationships that could be construed as a potential conflict of interest.

## Supplementary Material

The Supplementary Material for this article can be found online at http://www.frontiersin.org/Journal/10.3389/fonc.2013.00333/abstract

Figure S1 **Summary of disease progression events for patients with no, synonymous, and non-synonymous mutations in GRIN2A**. Data points for individual patients are shown; horizontal lines mark median values in each group. Levels of statistical difference between groups are shown. *Disease progression data for one patient with non-mutated GRIN2A were not available.

Table S1 **Summary of NZM cell lines tested in this study, together with their mutation status and systemic therapy received by patients prior to cell line establishment**.

## Footnotes

^1^http://primer3.wi.mit.edu/

^2^http://sift.jcvi.org/

^3^http://cancer.sanger.ac.uk/cancergenome/projects/cosmic/

^4^http://www.ncbi.nlm.nih.gov/projects/SNP
