# Supplementary material for: Evidence That GRIN2A Mutations in Melanoma Correlate with Decreased Survival
Source: Front Oncol. 2014 Jan 13;3:333. doi: 10.3389/fonc.2013.00333 (PMC3888952; doi:10.3389/fonc.2013.00333)
Supplement: Figure S1 — Summary of disease progression events for patients with no, synonymous, and non-synonymous mutations in GRIN2A. Data points for individual patients are shown; horizontal lines mark median values in each group. Levels of statistical difference between groups are shown. *Disease progression data for one patient with non-mutated GRIN2A were not available. [file 74132_Kalev-Zylinska_Presentation1.PDF]

Supplemental Figure S1. Summary of disease progression events for patients with no, synonymous and nonsynonymous mutations in *GRIN2A*. Data points for individual patients are shown; horizontal lines mark median values in each group. Levels of statistical diference between groups are shown. *Disease progression data for one patient with nonmutated *GRIN2A* was not available.

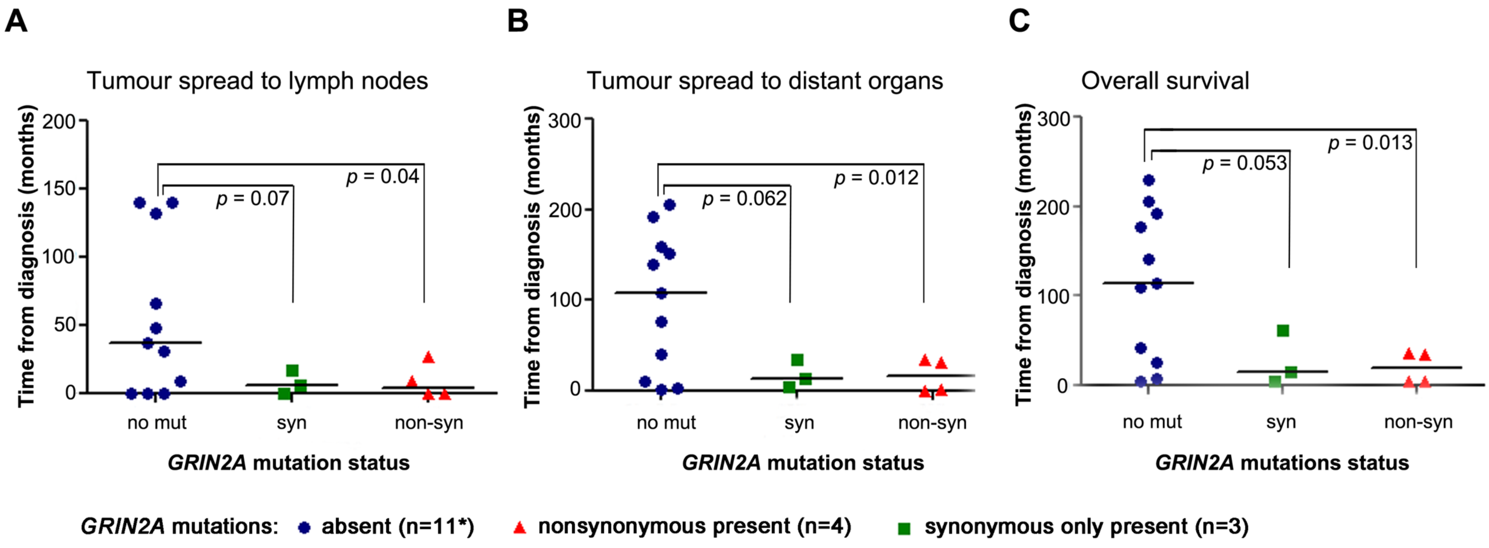

Supplemental Table S1: Summary of NZM cell line tested in this study, together with their mutation status and systemic therapy received prior to their establishment.

All patients were managed surgically and received radiotherapy to involved areas after tumour resection.

NZM cell line	Non-synonymous mutations in *GRIN2A*	Synonymous mutations in *GRIN2A*	*BRAF* V600E mutation	Treatment received prior to tumour resection
003	P1133S	F1344F	present	treatment naïve
007	G889E		present	treatment naïve
040		F186F	absent	Two cycles of POC chemotherapy completed 8 months prior to tumour resection. This was followed by a vascular-disrupting agent (6 months before) and Interferon α (2 months before resection)
055		L794L	present	treatment naïve
061	S349F; G762E	F177F	absent	treatment naïve
086		A1409A	absent	treatment naïve
100	P1132L		present	treatment naïve
004<br>006<br>011<br>020<br>030<br>034<br>076	absent	absent	present	treatment naïve
001<br>009<br>017<br>063<br>046	absent	absent	absent	Patient from whom NZM 017 was established received an autologous tumour vaccine a year before tumour resection.<br>Other cell lines were treatment naïve.
